# Making Waves: Why water reuse frameworks need to co-evolve with emerging small-scale technologies

**DOI:** 10.1016/j.wroa.2021.100094

**Published:** 2021-03-04

**Authors:** Eva Reynaert, Angelika Hess, Eberhard Morgenroth

**Affiliations:** a Eawag, Swiss Federal Institute of Aquatic Science and Technology, 8600 Dübendorf, Switzerland; b ETH Zürich, Institute of Environmental Engineering, 8093 Zürich, Switzerland

**Keywords:** On-site non-potable water reuse, Decentralized, Regulatory and legal frameworks, Guidelines, Standards, Field test

## Abstract

Novel technologies allow to reuse or recycle water for on-site applications such as toilet flushing, showering, or hand washing at the household- or building-scale. Many of these technologies have now reached technology readiness levels that require for verification and validation testing in the field. Results from such field tests of decentralized water reuse systems have been published over the past few years, and observed performance is often compared to quality targets from water reuse frameworks (WRFs). An inspection of ten recent journal publications reveals that targets from WRFs are often misinterpreted, and the emphasis of these publications is too often on demonstrating successful aspects of the technologies rather than critically evaluating the quality of the produced water. We hypothesize that some of these misinterpretations are due to ambiguous definition of scopes of WRFs (e.g., “unrestricted urban reuse”) and unclear applicability for novel recycling systems that treat the water for applications that go beyond the reuse scopes defined in current WRFs. Additional challenges are linked to the verification of WRF quality targets in small-scale and decentralized systems under economic and organizational constraints. Current WRFs are not suitable for all possible reuse cases, and there is need for a critical discussion of quality targets and associated monitoring methods. As the scope of water reuse has expanded greatly over the past years, WRFs need to address new applications and advances in technology, including in monitoring capacities.

## Introduction

Communities across the world face water supply challenges due to increasing demand, drought, groundwater depletion and contamination, dependence on single sources of supply, and ageing infrastructure (Hoffmann et al., 2020; Miller, 2006). In many regions, reclaimed water could be used as an alternative water source, as it provides reliable quantities of water, all while relieving the stress on freshwater resources (Hardy et al., 2015). Recent technological developments – especially the rapid development and wide acceptance of membrane technologies – have multiplied the potential applications of reclaimed water far beyond traditional reuse in agriculture (Angelakis et al., 2018; Chen et al., 2013). Novel small-scale technologies allow to reuse or recycle (i.e., reuse for the same application) water for on-site residential uses such as toilet flushing (Bair et al., 2015, Cid et al., 2018, Rogers et al., 2018, Sahondo et al., 2020), showering (Gassie and Engelhardt, 2017) or hand washing (Reynaert et al., 2020). Here, we use the term “small-scale” for technologies that operate at the household- or building-scale and are thus, by definition, decentralized. Especially for applications involving close contact to the water, such as hand washing or showering, the effective protection of user health is essential. Many of these non-potable water reuse technologies have now reached technology readiness levels that require verification and validation testing in the field (Mercer et al., 2018). Results from such field tests of small-scale water reuse and recycling systems have been published over the past few years.

To evaluate the microbial and chemical quality of the reclaimed water, many researchers refer to quality targets from legal and regulatory frameworks for water reuse (water reuse frameworks, WRFs), a term which is here used to designate regulatory tools such as guidelines, standards, regulations, laws, codes of practice, etc. To date, many countries have not issued national requirements for the quality of reclaimed water, especially for applications that go beyond agricultural reuse. Consequently, many WRFs are limited to reuse for irrigation, with the WHO guidelines for the safe use of wastewater, excreta and greywater in agriculture and aquaculture (WHO, 2006) as a widely-known example. For WRFs with focus on irrigation, a main goal is to protect the environment from high nutrient concentrations and other harmful substances, in addition to protecting human health. Increasingly, there are also WRFs targeting residential applications, with stronger emphasis on health-relevant parameters. For instance, the International Organization for Standardization has developed a standard for non-sewered sanitation systems, including performance requirements for unrestricted urban reuse (ISO 30500, ISO 2018). Sanz and Gawlik (2014) present an overview of WRFs around the globe and Reynaert et al. (2020) summarize requirements from 18 WRFs that are applicable to toilet flushing and handwashing reuse applications, including WRFs for the USA (US EPA, 2012), Western Australia (Government of Western Autralia, 2010), Canada (Health Canada, 2010) and Spain (Ministry of the Presidency Spain, 2007). For contexts for which no WRFs exist, environmental technology verification (ETV) programs have been developed to verify claims about the performance of innovative environmental technologies by qualified third parties.

An inspection of field testing literature revealed that there is quite a range of approaches on choosing the appropriate WRF for small-scale residential applications and how to interpret the quality targets from such frameworks. This paper identifies the main errors and analyses whether these are due to inherent problems of the WRFs or to an inappropriate application. We propose measures to avoid erroneous interpretations of quality targets, describe how WRFs need to evolve with technology advancement and outline future research needs. While we do not evaluate water reuse technologies nor present specific suggestions on how to define WRF quality targets in this paper, we hope to initiate a discussion on such targets and associated monitoring methods amongst water reuse experts from science and practise.

## Common misinterpretations of water reuse frameworks applied to novel small-scale water reuse technologies

We investigated ten recent publications (including our own) on field tests of water reuse systems that compare their monitoring results to quality targets from WRFs (Reynaert et al., 2020; Sahondo et al., 2020; Varigala et al., 2020; Welling et al., 2020; Cid et al., 2018; Hawkins et al., 2018; Rogers et al., 2018; Fountoulakis et al., 2016; Santasmasas et al., 2013; Knerr et al., 2011). The criteria for including a publication were: (i) water reuse system with the aim for unrestricted urban reuse (e.g., toilet flushing); (ii) field test or full-scale test with real wastewater input; and (iii) comparison of reclaimed water quality to targets from WRF. These publications were analysed in terms of selection, application and interpretation of WRFs, as summarized in Table 1 (detailed evaluation in supplementary information Table S.1, doi:10.25678/0002QD, including details on WRFs used). The purpose of this evaluation is not a judgement on research quality in these publications, but to highlight the challenges of linking novel reuse technologies and applications with appropriate WRFs.

**Table 1:** Evaluation of ten publications on field tests or full-scale tests (using real wastewaters) of household- or building-scale water reuse technologies illustrating common misinterpretations of water reuse frameworks (WRFs). Full details of the publications are included in supplementary information Table S.1 (doi:10.25678/0002QD). Shading in column “publications with incorrect assessment” indicates incorrect percentage: green <25%, yellow between 25% and 50%, orange > 50%.

**Selection of WRFs:** To allow for a meaningful interpretation, a WRF must be locally applicable, adapted to the type of wastewater input (e.g., grey- or blackwater), and include adequate reuse purposes (e.g., toilet flushing). All but two publications referenced the water reuse or discharge frameworks unambiguously, and all but one applied either locally-relevant (when existent) or international WRFs, or both. With one exception, all publications used WRFs that were adapted to the type of wastewater treated. Most errors were linked to the selection of WRFs that do not cover the intended application scope: only one out of nine publications selected (only) quality targets aimed at the intended reuse application, while six publications (also) compared their monitoring results to quality targets for agricultural reuse or surface water discharge, though they examine systems which are intended for unrestricted urban reuse (e.g., reuse as toilet flush water). This is problematic, because the logic behind the quality targets may differ (health-based considerations vs. protection of the environment).

**Application of WRFs:** The application of WRFs was evaluated in terms of consistency. Only two out of ten publications unambiguously defined all quality targets. Four publications did not compare all parameters that were measured during the studies and for which quality targets were defined in the applied WRFs. Another issue was the incomplete application of different WRFs (in addition to a main reference WRF) only for selected parameters, rather than all parameters defined in the WRFs. We hypothesize that these inconsistencies were due to a selection of WRFs after the testing, e.g., the frameworks were selected to meet the field test design instead of designing the testing to match the framework specifications. The evaluation of the correct interpretation of the WRFs was hindered in many publications, as they did not provide sufficient information for the reader to be able to compare the monitoring results to the quality targets. Many errors were linked to an inaccurate interpretation of quality targets from WRFs. WRFs set different types of quality targets, which in some cases may be simple maximum, minimum or average concentrations. In many cases, however, they set more complex targets (e.g., effluent requirements in ISO 30500: achieve quality targets in 4 out of 5 events, with no more than 20% variance from the target for any failed parameter), which require for an adequate testing design. Many publications presented average values for the monitoring parameters, when the quality targets were defined as maximum or minimum values, leading to a misinterpretation of their testing results.

**Interpretation of WRFs:** The evaluation of the quality targets is often described in the abstract or in the conclusion of the publication and is used as an argument in favour of the researched technology. Because an extended representation of the results is not possible in these sections, the results are sometimes simplified to a point that is no longer correct, which is the case in seven out of nine publications. Examples of such oversimplified statements are “nearly met” or “partially met”. Such statements show that too often the focus lies on the success of the technologies rather than on a critical discussion of the water quality reached. When the researched technology and the target reuse application are not covered in the WRF, it is of great importance to critically assess the suitability of the given quality targets. Only one publication critically discussed these mismatches.

## On the difficulty of applying water reuse frameworks for small-scale water reuse applications

An investigation of the challenges involved in the establishment of WRFs can help understanding some of the widespread issues with their correct application and interpretation to novel water reuse systems. Most countries worldwide have adopted national regulations or standards for drinking water quality (WHO, 2018), with a focus on the protection of human health. Generally, centralized solutions are favoured for the supply and distribution of the water (Peter-Varbanets et al., 2009), which allows for the use of laboratory-based analytical methods for (microbial) quality control. The establishment of WRFs involves increased complexity. Especially for novel and small-scale technologies, this can translate into difficulties in the correct application of WRFs. Reasons are:

1. Diverse water inputs (e.g., greywater vs. blackwater vs. mixed wastewater) and a wide range of reuse applications (e.g., irrigation vs. handwashing). This can result in ambiguous application scopes, with many WRFs defining broad reuse categories such as “unrestricted urban reuse” (US EPA, 2012) or “supply to sanitary appliances” (Spanish regulation RD 1620/2007 translated by the Universitat Politècnica de Catalunya (2011), which cover a wide range of potential reuse applications with tremendous differences in human health risks.
2. The necessity to incorporate human health as well as environmental considerations. The interpretation of quality targets is particularly difficult for recycling systems without discharge to the environment. Many WRFs set effluent quality targets that aim at the protection of the environment (e.g., phosphorus removal ≥ 80% according to ISO 30500), but it is not clear whether meeting such targets is required for systems that fully recycle the water.
3. As an additional difficulty, water reuse technologies are increasingly small-scale and decentralized (Daigger and Crawford, 2007). For such systems, laboratory-based methods may no longer be suitable due to economic and organizational constraints, making adequate monitoring of the water quality another challenge. To this day, however, most WRFs require frequent measurements of multiple water quality parameters. As an example, the US EPA (2012) recommends continuous measurements of turbidity and chlorine, daily monitoring of faecal coliforms and weekly measurements of pH and biological oxygen demand. It is unclear how such a monitoring system and scheme can be economically implemented in a small-scale (e.g., household-based) water reuse system. Monitoring of many of these parameters requires access to well-equipped laboratory facilities, which may not be available in many contexts in which alternative water sources are most needed.

In our own work (Reynaert et al., 2020), we faced all of these challenges when field-testing prototypes recycling handwashing water in Switzerland and South Africa: (1) It was not clear to what extent categories such as “unrestricted urban reuse” or “supply to sanitation appliances” were intended to cover high-quality (and thus high-risk) applications such as handwashing. Only one of the applicable WRFs explicitly mentioned handwashing (ISO 30500, 2018), however, without providing explicit quality targets. (2) We were unsure to what extent the target (effluent) requirements were applicable to our closed-loop water recycling without effluent to the environment. (3) We had to transport samples over a distance of almost 50 km for analysis and the cost of the implemented online monitoring system surpassed the cost projection for the industrialized water reuse system.

We believe that some of the misinterpretations presented in Table 1 could have been avoided with more critical scrutiny by the authors. Still, these examples illustrate that many WRFs were not developed for the novel small-scale water reuse technologies that are being field tested now, hindering their judicious application.

## Way forward

Research on water reuse technologies is societally and environmentally highly relevant and receives increasing attention from industrial manufacturers and utilities operators. Longer term, many of the novel water reuse technologies aim towards industrialization and commercialization. From our own experience, we know that publications on novel water reuse technologies are read by a varied audience, including readers that do not have a background in engineering and may not be familiar with WRFs. Especially in such a context, it is critical to introduce and apply quality targets with increased diligence.

Best practice principles are needed for a careful and transparent use of WRFs. Carefulness comprises (i) the selection of a locally-relevant WRF before the testing, so that appropriate monitoring parameters and methods can be selected, (ii) having an understanding of the WRF (including general testing and monitoring requirements) that goes beyond the quality target values, and (iii) using WRF quality target values as a reference point rather than a goal (e.g., avoiding statements like “mostly/nearly met quality targets”). Transparency requires (i) the full details of the selected WRF, including a justification on why it was selected, (ii) an explicit mention of the parameters that are defined in the WRF, but were not measured in the study, and (iii) a presentation of the results in a form that the reader can compare them to the target values.

But researchers have to think further than these best practice principles, as technology and regulations need to be co-evolving. Researchers should go beyond a conscientious evaluation of the water quality and engage in the critical discussion of quality targets and associated monitoring methods from WRFs. Quality targets are tools that were developed to ensure the protection of human and environmental health. These quality targets will vary with scientific and technological development, economic and environmental constraints, and cultural and political shifts (Hespanhol and Prost, 1994). Thus, it is not sufficient to select and implement the correct WRF, where the WRF may not have been aware of the technology options available. For an effective protection of human health and the environment, we advocate for the implementation of WRFs that use risk-based (and thus end-use dependant) system performance targets, for instance based on quantitative microbial risk assessments. We see a responsibility of researchers to not blindly follow quality target values, but critically assess the suitability of these targets for their specific testing context and technology and thus contribute to the further development, revision and expansion of frameworks for water reuse. This is particularly needed for

1 novel technologies that allow the reuse of water for applications that are not covered in existing frameworks
2 recycling technologies that do not produce an environmental effluent
3 applications with increased health-risks for the users (e.g., direct contact with the water), where online monitoring of the hygienic water quality becomes indispensable

Ultimately, water reuse technologies will not be certified or approved based on the field studies presented here, as this is the responsibility of regulatory authorities or independent certification organizations. But during field testing, researchers are confronted with real-world problems such as lacking access to a laboratory. Field testing thus not only provides an opportunity to validate technologies, but also their maintenance, control, and monitoring of the reclaimed water quality. With this experience, the goal of researchers should not be to fulfil the WRF targets but also to make use of their experience and knowledge to critically discuss the WRFs. This critical discussion is a valuable feedback that allows the WRFs to co-evolve with emerging technologies. These evolved WRFs will provide reasonable guidance to operators of conventional systems and therefore to ensure protection of the environment and public health.

## Conclusions

• Quality targets from WRFs are often misinterpreted. While some misinterpretations are avoidable if applying due diligence, many WRFs are only partially applicable to novel small-scale water reuse systems.
• WRFs and associated monitoring strategies need to co-evolve with advances in technology and new applications.
• Field testing of novel technologies provides an opportunity to test the applicability of WRFs. This evaluation is only possible if researchers do not only focus on the success of the technology (i.e., showing which quality targets are met), but also consider the frameworks’ overarching principles.
• An effective policy and regulatory process to provide adequate treatment of water for the intended end use will be key to the viability of small-scale and decentralized water reuse systems.

## CRediT authorship contribution statement

**Eva Reynaert:** Conceptualization, Formal analysis, Writing - original draft, Writing - review & editing. **Angelika Hess:** Conceptualization, Formal analysis, Writing - review & editing. **Eberhard Morgenroth:** Conceptualization, Writing - original draft, Writing - review & editing.

## Declaration of Competing Interest

None.
